# A Schatzker Type III Tibial Plateau Fracture in a Soccer Player: A Case Report

**DOI:** 10.7759/cureus.42015

**Published:** 2023-07-17

**Authors:** Kevin Do, Alan A Zakaria, Tais G. O Bertasi, Raphael A. O Bertasi, Rock P Vomer, Jeffrey Nadwodny, George G. A Pujalte

**Affiliations:** 1 Family Medicine, Trinity Health, Lake Orion, USA; 2 Sports Medicine, Beaumont Hospital, Troy, USA; 3 Internal Medicine, Mount Sinai Morningside West, New York, USA; 4 Family Medicine, Mayo Clinic, Jacksonville, USA; 5 Family Medicine and Community Health, Orthopedics, Division of Sports Medicine, Duke University, Durham, USA

**Keywords:** operative management, trauma, tibial injury, tibial fracture, conservative management

## Abstract

The tibial plateau is an important load-bearing surface in the knee, and when fractured, there is subsequent loss of motion and stability. These fractures typically result from axial loading and twisting. Our case outlines a tibial plateau fracture in a 15-year-old soccer player. The physical examination was positive for a decreased range of motion, pain with valgus stress, and positive ballottement. Radiography of the knee revealed joint effusion but no definite fracture. MRI revealed a Schatzker Type III fracture and a partial medial collateral ligament (MCL) tear. Our patient was referred for open repair and internal fixation. The Schatzker classification system is divided by type and location of fracture. Types I through III are located laterally, Type IV is medial, Type V identifies bicondylar fractures, and Type VI identifies tibial diaphysis separation from the metaphysis. These fractures are managed both nonoperatively and operatively. Nonoperative management is recommended for minimally displaced fractures that will heal without notable deformity. Operative management is indicated for displaced and unstable fractures, which include all fracture Types IV through VI, and certain Type I through III fractures that have valgus alignment or large articular surface involvement. Recovery time is lengthy and largely dependent on the fracture type. The first six weeks usually involve non-weightbearing, the second six weeks include weightbearing as tolerated, with knee range of motion exercises and muscle strengthening. Although open repair and internal fixation usually provide good results, some athletes cannot return to their previous levels of activity.

## Introduction

The tibial plateau is a critical load-bearing surface in the knee; disruption results in malalignment, instability, and functional deficits. Overall, tibial plateau fractures constitute approximately 1% of all fractures [1]. In elderly patients, it comprises up to 8% of all fractures [2]. Timely recognition and treatment are imperative for minimizing disability and deformity and reducing adverse effects. The mechanism of injury is axial loading from falls, twisting, or valgus force [1]. These fractures can present with associated injuries to the lateral meniscus, medial collateral ligament (MCL), anterior cruciate ligament, fibular neck or head, or peroneal nerve [1].

## Case presentation

A 15-year-old soccer player presented to our clinic with right knee pain three days after an in-game collision that resulted in immediate pain, swelling, and removal from the game. He denied hearing a “pop” or experiencing any locking or catching. The pain was over the medial knee. Upon examination, the right knee was swollen, with joint line tenderness and decreased range of motion. The pain was produced with valgus stress testing, with no laxity. There was no patellar tendon tenderness. Lachman, anterior drawer, patellar apprehension, McMurray, and posterior drawer tests were negative. Initial differential diagnoses included MCL sprain, meniscus tear, femoral condyle fracture, and tibial plateau fracture.

Radiography of the right knee revealed a small effusion with no definite fractures or dislocations. MRI revealed a 4-mm depressed fracture involving the anterior one-third of the lateral tibial plateau, compatible with a Schatzker Type III fracture, and a partial MCL tear.

## Discussion

We present the case of a 15-year-old soccer player with a Type III tibial plateau fracture and a partial tear of the MCL. Five to 10% of all tibial plateau fractures are sports-related, and proximal tibial fractures are common in high-energy sports [1].

The decision to manage a tibial plateau fracture conservatively depends on predicting the likelihood of healing without deformity. Cutoffs for displaced or depressed fractures with a step-off to undergo open reduction and internal fixation (ORIF) varies [3]. The current indications for operative treatment of a lateral plateau fracture having more than a 3-mm articular step-off, more than 5 mm of condylar widening, and/or varus or valgus instability. Moreover, displaced medial plateau fractures, bicondylar fractures, having signs or symptoms of neurovascular compromise, and open fractures, are treated operatively [4]. 

Typically, it takes about one year to recover from a tibial fracture [3] and, within that timeframe, around 70%-90% of individuals are able to return to work [5]. Post-traumatic arthritis is a common adverse effect and most commonly occurs when there is cartilage damage [6].

**Figure 1:**
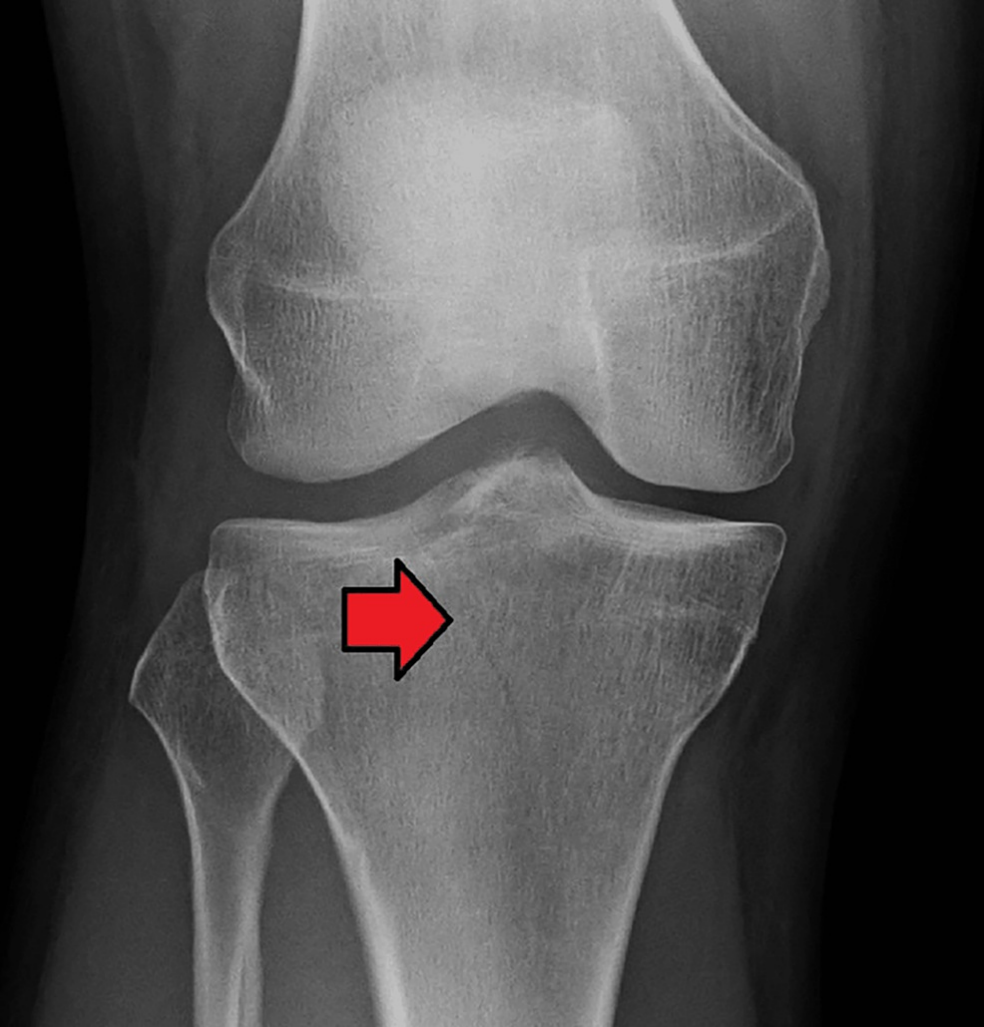
Anteroposterior view radiograph in plane of the plateau showing a subtle, Schatzker Type I tibial plateau fracture (arrow).

Physical findings of tibial plateau fractures can be subtle. The diagnosis can be made with radiography (anteroposterior, lateral oblique, and optional plateau views) (Figure 1) [3].

The plateau view improves the visualization of displacement and depressions [3].

CT can define the location of fragments, fracture lines, and the size of articular segments [3]. MRI is used to locate fracture lines, degree of articular displacement, and injuries to menisci and ligamentous structures. MRI can identify meniscal and ligamentous pathologies, and occult fractures more effectively than radiography and is equivalent to traditional CT. Therefore, when a proximal tibial stress fracture is suspected MRI is preferred [3]. Yacoubian et al. showed that CT may change the fracture classification type up to 6% when compared to radiography alone, while MRI can change it up to 21%, thus MRI is important for the treatment plan [7].

Classification of tibial plateau fractures

**Figure 2:**
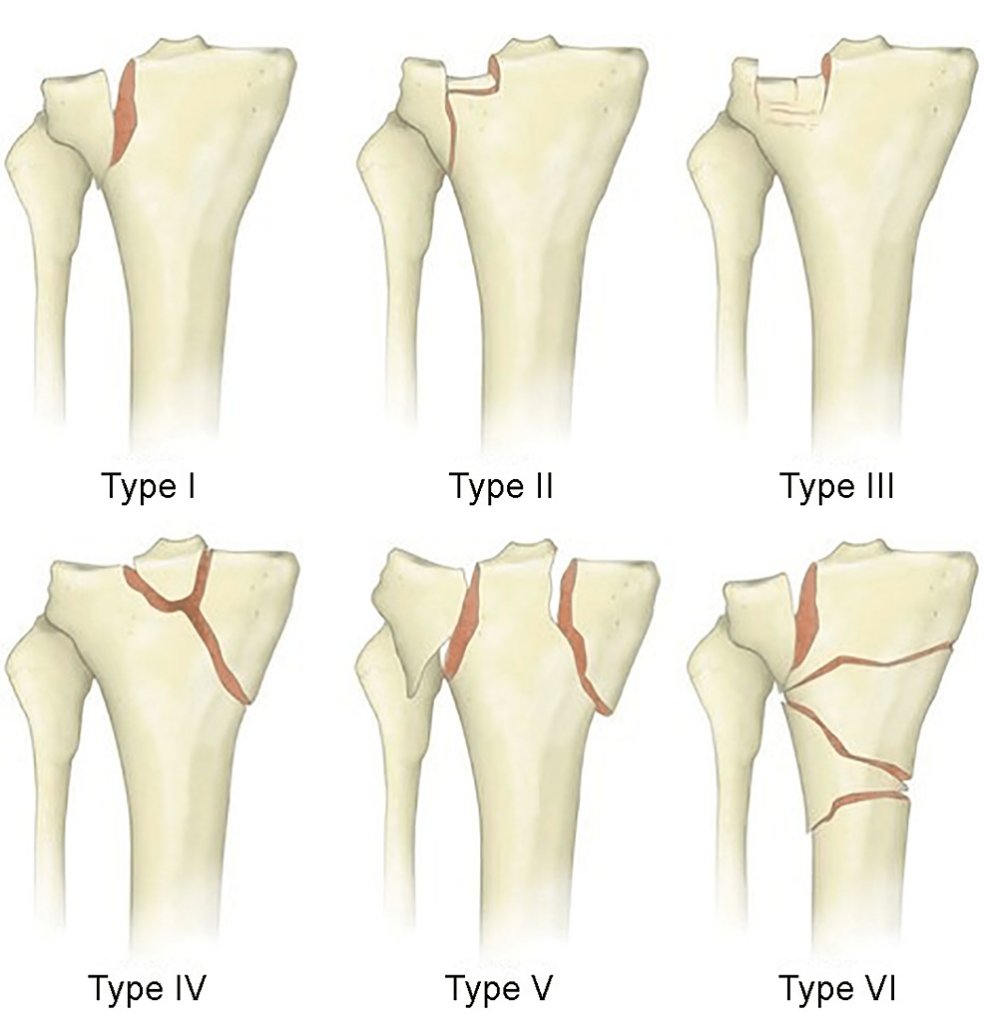
Schatzker classification of tibial plateau fractures. Previoulys published by Em Docs, http://www.emdocs.net/em3am-tibial-plateau-fracture/. Used under the terms of the Free Open Access Meducation (FOAMed) initiative.

The most accepted fracture classification system is the Schatzker classification [3], which defines fractures by pattern and fragment anatomy. Types I through III comprise lateral-sided fractures, Type IV identifies medial-sided fractures, and Type V identifies bicondylar fractures. Type VI represents metaphyseal-diaphyseal disassociation, with significant soft-tissue injury (Figure 2).

Treatment and management

**Figure 3:**
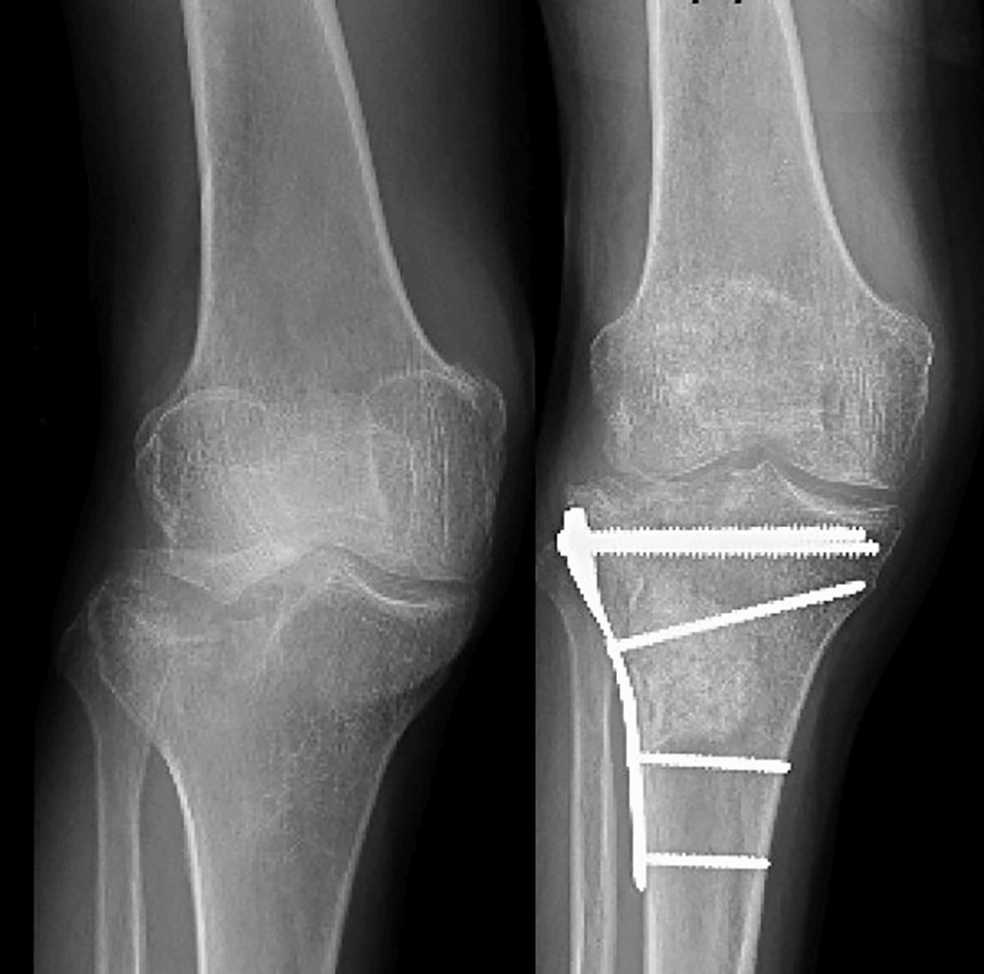
A Schatzker Type III fracture after ORIF with lateral plate and screws. Previously, published in https://epos.myesr.org/poster/esr/ecr2015/C-1752. Used under the terms of publisher’s conditional open license [4]. ORIF, open reduction and internal fixation

Our patient was referred to orthopedics for ORIF (Figure 3).

The decision to manage nonoperatively or operatively depends on fracture configuration, concomitant soft tissue injury, and patient age, activity level, and bone quality [1].

Conservative management is recommended for nondisplaced or minimally displaced fractures that will heal without notable deformity, poor surgical candidates, and patients who would not benefit functionally [3]. Predicting the likelihood of deformity after treatment is imperative, as angular deformity increases the weightbearing load on the most damaged portion of the articular surface and is not well tolerated. Cast braces are rarely used to offload the injured side of the joint; patients are kept from weightbearing activities for four to eight weeks. Knees tolerate up to six weeks of immobilization before stiffening; this is prevented with non-weightbearing mobilization in a hinged brace [3].

Operative management is preferred for unstable displaced fractures [8]. Operative treatment is for Type V and VI fractures, most Type IV fractures, and Type I through III fractures where valgus alignment is imminent without fixation [3]. For Type I through III, the presence of a split fragment, depression involving half of the lateral articular surface, fibular head fracture, or clinical or radiographic valgus alignment are indications for surgery. In older, less active, or medically unfit patients, operative management is determined case by case [3].

Studies comparing ORIF to arthroscopy-assisted percutaneous internal fixation have shown no significant difference in duration of operation, fracture healing time, or postoperative flexion [1, 9]. However, arthroscopy-assisted percutaneous internal fixation may achieve anatomic reduction after surgery in a greater number of patients, easier and more rapid postoperative rehabilitation, and earlier weightbearing [1].

Tibial plateau fractures have a high rate of concomitant ligament and meniscal injuries. Conservative management of MCL injuries is generally favored; most do not have long-term sequela, and residual laxity usually relates to bony depression rather than ligament laxity [3]. Management of other associated intra-articular soft tissue injuries is controversial. Some authors recommend aggressive management, while others recommend a more conservative approach [3]. Meniscal injuries associated with tibial plateau fractures often have positive outcomes without surgical intervention as most of them do not lead to long-term limitations [4]. Reattaching avulsed anterior or posterior cruciate ligaments, when associated with a fracture fragment following a high-energy injury, is recommended [3].

Postoperatively, six to 12 weeks of non-weightbearing is required, determined by fracture pattern and type of fixation. An early unloaded range of motion is important to prevent joint stiffening. Structured therapy should be used to reintroduce progressive weightbearing only after the non-weightbearing period.

## Conclusions

Occult tibial plateau fractures require a high index of suspicion and they should be investigated with a CT scan or an MRI. ORIF of lateral tibial fractures provides acceptable clinical outcomes. Postoperatively, non-weightbearing and early mobility are important to prevent fracture displacement and maintain knee motion. Although operative management provides good results, some athletes are not able to return to their previous level of activities.
